# Multivariate trait analysis reveals diatom plasticity constrained to a reduced set of biological axes

**DOI:** 10.1038/s43705-021-00062-8

**Published:** 2021-10-25

**Authors:** Phoebe A. Argyle, Nathan G. Walworth, Jana Hinners, Sinéad Collins, Naomi M. Levine, Martina A. Doblin

**Affiliations:** 1 Climate Change Cluster, University of Technology Sydney, Sydney, NSW 2007 Australia; 2 Department of Biological Sciences, University of Southern California, Los Angeles, CA 90089-0371 USA; 3 Institute of Coastal Ocean Dynamics, Helmholtz-Zentrum Hereon, 21502 Geesthacht, Germany; 4 Institute of Evolutionary Biology, University of Edinburgh, Edinburgh, EH9 3JF UK; 5 Sydney Institute of Marine Science, Mosman, NSW 2088 Australia

**Keywords:** Microbial ecology, Theoretical ecology

## Abstract

Trait-based approaches to phytoplankton ecology have gained traction in recent decades as phenotypic traits are incorporated into ecological and biogeochemical models. Here, we use high-throughput phenotyping to explore both intra- and interspecific constraints on trait combinations that are expressed in the cosmopolitan marine diatom genus *Thalassiosira*. We demonstrate that within *Thalassiosira*, phenotypic diversity cannot be predicted from genotypic diversity, and moreover, plasticity can create highly divergent phenotypes that are incongruent with taxonomic grouping. Significantly, multivariate phenotypes can be represented in reduced dimensional space using principal component analysis with 77.7% of the variance captured by two orthogonal axes, here termed a ‘trait-scape’. Furthermore, this trait-scape can be recovered with a reduced set of traits. Plastic responses to the new environments expanded phenotypic trait values and the trait-scape, however, the overall pattern of response to the new environments was similar between strains and many trait correlations remained constant. These findings demonstrate that trait-scapes can be used to reveal common constraints on multi-trait plasticity in phytoplankton with divergent underlying phenotypes. Understanding how to integrate trait correlational constraints and trade-offs into theoretical frameworks like biogeochemical models will be critical to predict how microbial responses to environmental change will impact elemental cycling now and into the future.

## Introduction

Trait-based approaches to ecology focus on how an organism’s physical, chemical, and physiological characteristics (traits) contribute to its population size and dynamics [1]. Traits are often used to simplify interactions between organisms and their environment and the resulting impact on community and ecosystem level function, with less focus on specific taxonomic diversity, i.e., studying individual species separately [2]. These approaches, originally developed in plant biology, have been extended to many organisms, including fungi, invertebrates, fish, and birds [3–6]. Seminal work in ecology has used relationships between traits to understand fundamental ecological principles, for example Metabolic Scaling Theory [7–9].

The application of trait-based approaches to phytoplankton has gained traction in the last two decades [10–13]. Phytoplankton play crucial roles in global elemental cycles and are often classified into functional groups (e.g. silicifiers, calcifiers, nitrogen-fixers) based on traits that define their roles in global elemental cycles, including carbon (C), phosphorus (P), nitrogen (N), and silica (Si) cycles [14]. Using trait-based functional groups simplifies the high taxonomic and morphological diversity of phytoplankton and allows the incorporation of traits such as growth, nutrient uptake, efficiency of light utilisation, and temperature tolerances into biogeochemical and ecological models [15–18].

These models utilise a large body of literature which seeks to identify potential trade-offs between traits, where organisms increase functionality or efficiency in one aspect of their physiology at the cost of another [10, 19–21]. These trade-offs are usually studied as pairwise comparisons, based on the relationship between trait values, with negative correlations indicating a potential trade-off. The observed correlative relationships between traits often derive from more complex causal dynamics. For example, a unimodal relationship between size and growth rate is observed for marine microbes and used to parameterize biogeochemical models [18, 22]. This relationship between size and growth rate emerges due to a trade-off between nutrient acquisition and maximum metabolic rate [23], though other trade-offs also affect the size and growth rate relationship [24], which highlights the need to consider more than pairwise correlations. Beyond pairs of traits, there is also evidence for three-way trait trade-offs, for example between nitrogen and phosphorus nutrient uptake at low concentrations and cell size [25].

Furthermore, trait-based models rely on fixed trait relationships derived from interspecific variation [25–29]. As such, these models do not capture the high level of trait variation both between and within species of a functional group [30–33]. There is growing evidence that substantial intraspecific variation has the potential to play a key role in the evolutionary responses of species to environmental change [1, 31, 32, 34]. By not considering phenotypic plasticity within a population or species, existing biogeochemical models are lacking a critical mechanism underlying the adaptive response of phytoplankton to environmental change and therefore may produce biased predictions of future changes.

While the incorporation of phenotypic plasticity into biogeochemical models is needed, this is a daunting task due to the complexity of the problem. An improved understanding is needed as to the breadth of intraspecific trait variation, correlations between traits and their plastic responses, the degree of phenotypic plasticity within phytoplankton functional groups, and constraints on this plasticity. It is experimentally intractable to rigorously test all possible trait combinations across many phytoplankton lineages and under a range of environmental conditions. Thus we need to develop an understanding of how traits are interrelated through multi-dimensional trait-based approaches. Due to trade-offs and correlations between traits, it has been hypothesised that variability over many traits can be captured in a reduced number of underlying axes [35]. Such a set of reduced dimensionality trait-axes has been demonstrated for a soil microalga *Chlamydomonas* [35] but how this pattern generalizes to phytoplankton is yet to be investigated. If such a set of reduced axes of variance can be defined, phenotypic variation and incorporating the impact of this variability becomes computationally tractable [34, 35].

In this study, we test the idea that when complex phenotypes (>3 traits) are measured, traits co-vary and thus constrain the variability of observed phenotypes. We used multivariate analyses that emphasize the relationships between traits to examine patterns in inter- and intraspecific phenotypic variability and plasticity in the cosmopolitan diatom genus *Thalassiosira*. Diatoms contribute significantly to C and Si cycling in the oceans, due to their contribution to global primary productivity (~20%; [24]) and their deposition of frustules [27, 36]. We measured 9 functional traits in 13 strains representing 7 species. These traits were chosen to cover basic physiological functions (e.g., light utilisation efficiency) and we included several traits that are directly relevant for biogeochemical function (e.g., population growth, cell size).

We used principal component analysis (PCA) to create a two-dimensional ‘trait-scape’ [34] that allows us to place complex phenotypes on a reduced number of axes (dimensions) and to uncover underlying patterns of variability between strains or species. The majority of the variability between phenotypes was described by two main orthogonal axes within the 2-dimensional trait-scape. Due to covariance between the traits, we found that the same trait-scape could be recovered using a subset of input traits, highlighting that the underlying patterns of variation can be uncovered without using exhaustive numbers of measurements. The parameterization of multidimensional trait variation represents a significant challenge [27], and the use of PCA with a subset of traits that are reasonably easy to measure is one method for addressing this challenge.

Genetic/taxonomic differences between *Thalassiosira* strains could not predict phenotypic diversity within the trait-scape, and phenotypes resulting from plastic responses to two new growth environments were more divergent than between-strain differences. However, consistent over-arching “strategies” were identified when strains were exposed to new environments, indicating that changes in phenotype due to plastic responses are constrained and follow a pattern.

These findings highlight the utility of multi-trait approaches for observing patterns and constraints on both standing trait variation and plastic responses to environmental change in phytoplankton that in turn could be used to anticipate how phytoplankton traits may shift in future climate scenarios.

## Materials and methods

### Culture maintenance and growth

Twelve strains of *Thalassiosira* spp. were obtained from the Provasoli-Guillard National Centre of Marine Phytoplankton (NCMA, https://ncma.bigelow.org/), and one strain from the Australian National Culture Collection, representing 7 species in total (Supplementary Table 1). Cultures were maintained in polystyrene tissue culture flasks in artificial seawater with f/2 media [37] at 20 °C, with 60 µmolm^−2^s^−1^ of light on a 12:12 light cycle.

Three strains originally identified as *Thalassiosira* sp. in the NCMA collection were further classified to the species level using sequencing of the ITS2 gene region (Supplementary Table 1): CCMP1055 as *T. auguste-lineata* (84.64% similarity; [38]) and CCMP2929 as *T. weisflogii* (98.37% similarity to Strain 1587 used in our study; [39]). Strain CCMP1059 was tentatively identified as *Cyclotella striata* (94.17% identity match to clone ZX28-3-40; [40]) also from order Thalassiosirales, but this assignment requires further investigation.

### Experimental set up

Experimental cultures (200 mL) were grown in 250 mL polystyrene tissue culture flasks in triplicate, at a starting concentration of 2500 cells ml^−1^. All 13 strains were grown in a “standard” environment (identical to maintenance conditions) with 9 phenotypic traits measured to describe the initial trait-scape. Five strains (1010, 1059, 2929, 3264, and 3367) were grown in two additional environments in triplicate: a high temperature and light treatment (HT: 30 °C, 200 µmol photons m^−2^s^−1^ of light, 12:12 light:dark), and a low nutrient treatment (LN: f/400 media with an adjusted N:P ratio of 10:1 achieved by reducing the nitrate concentration from 4.4 to 1.8 µM, 60 µmol photons m^−2^s^−1^ of light, 12:12 light:dark). Cultures for the two additional treatments were inoculated with 10,000 cells ml^−1^ (LN) and 5,000 cells ml^−1^ (HT) in anticipation of limited growth.

Growth was tracked daily using in vivo fluorescence as a proxy for cell density [41]. One mL aliquots of experimental cultures were measured for chlorophyll-a fluorescence using a plate reader (TECAN Infinite M1000 Pro, Männedorf, Switzerland) using 455/680 nm excitation/emission spectra. Phenotypic traits were measured at mid-late exponential phase, assessed by visually examining in vivo fluorescence growth curves. In the case of the low nutrient treatment, where growth was limited to 3–5 days, cultures were harvested in early stationary phase. Duration of growth for each experiment is summarised in Supplementary Table 2.

### Phenotypic trait measurement methods

Phenotypic traits were selected to capture different commonly measured base physiological functions, and to include traits that are used in biogeochemical models. We also selected traits that demonstrated independence and orthogonality (i.e., not all co-varying), based on pilot studies, in order to successfully define the multivariate trait-scape [42].

#### Growth rate

Growth rates for each time step were calculated from the daily in vivo fluorescence measurements according to the calculation:

$$\mu = \frac{{{{{{{{{\mathrm{ln}}}}}}}}\left( {F_2} \right)-{{{{{{{\mathrm{ln}}}}}}}}\left( {F_1} \right)}}{{t_2 - t_1}}$$

Maximum growth rates were determined by the average growth over 2–4 consecutive steps depending on the duration of exponential growth.

#### Flow cytometry traits

For flow cytometry trait measures (growth rate, size, chlorophyll a content, lipid content), 1 mL aliquots of experimental culture were fixed with EM grade paraformaldehyde (0.8% final concentration, Electron Microscopy Sciences, Ft Washington, PA) in 1.6 mL cryopreservation tubes (CryoPure, Sarstedt), frozen in liquid nitrogen, then stored at −80 °C prior to analysis. All measures were performed using a Cytoflex LX (Beckman Coulter, CA, USA).

#### Cell counts and size

Cell counts were done by gating the diatom population using chlorophyll a (488 nm excitation, 690/50 nm detector) and forward scatter channel thresholds. Cell size was estimated using forward scatter values calibrated against spherical beads (2, 4, 6, 10, 15 µM diameters; Invitrogen, CA). This resulted in a conversion equation of equivalent spherical diameter (ESD) = (FSC + 194636)/75775, which was used to assess relative changes in cell size [43].

#### Chlorophyll a content

Chlorophyll a (Chl-a) fluorescence of the gated diatom population was quantified using 488 nm excitation, 690/50 nm detection. A standard bead (Cytoflex Daily QC Fluorospheres; Beckman Coulter) was used to calibrate the performance of the instrument and ensure comparable measures across samples. Chlorophyll values were divided by ESD to account for cell size differences.

#### Side scatter/granularity

Side scatter is an indicator of the internal complexity of a cell or “granularity”. This trait is measured in tandem with other flow cytometry measures and was included as a phenotypic trait. The interpretation of this trait is not straight forward, but is independent of other flow cytometry traits measured and has been used in other flow cytometry studies of microalgae [44]. This trait was divided by ESD to account for cell size differences.

#### Neutral lipids

Relative neutral lipid content was determined using the fluorescent stain BODIPY™ 505/515 (Thermo Fisher, MA, USA) which is commonly used to assess neutral lipid content in phytoplankton [45–47]. Background fluorescence (488 nm excitation, 525/40 nm detector) of PFA-fixed cells was measured in tandem with the size, chlorophyll a, and side scatter. After this, 10 µL of BODIPY stain (2 mg mL^−1^ in DMSO) was added to each sample, resulting in a final BODIPY concentration of 2 μg mL^−1^. Samples were incubated for 10 min in the dark before being read again on the flow cytometer. Neutral lipid content was defined as the difference in median fluorescence per cell between the pre- and post-stained sample. This value was then divided by the ESD size to account for size-related effects.

#### Photophysiological traits

Photophysiological measures were taken by conducting a rapid light curve [48] with a water PAM (Water-PAM; Walz GmbH, Effeltrich, Germany) using 1 mL of experimental culture diluted in artificial seawater. The rapid light curve protocol exposes the culture to 8 steps of increasing irradiance for 10 seconds each, measuring the photophysiological response at each step. Maximum electron transport rate (ETRmax), Ik (half saturation irradience), and alpha (the photosynthetic rate during the light-limited linear region) were calculated using the regression fit function in the PAM WinControl software. Photophysiology measurements were taken between 4–5 h after the start of the photoperiod.

#### Reactive oxygen species

The development of reactive oxygen species (ROS) was measured using the fluorescent probe 2’,7’-dichlorodihydrofluorescein diacetate (H_2_DCFDA; Thermo Fisher, MA, USA) which has been used in a number of phytoplankton studies [49–51]. Two 1 mL aliquots of experimental culture were transferred to a 48 well tissue culture plate; 2 µL of stain (2.5 mg mL^−1^ H_2_DCFDA was made in DMSO) was added to one aliquot, with the other acting as a blank. The plates were sealed (Breathe-Easy, Diversified Biotech) and incubated in the dark at growth temperature (20 or 30 °C) for 2 h. Incubation was done in the dark because of the effects of light on the dye itself, therefore the effects of the excess light treatment were not captured in this trait. Fluorescence of H_2_DCFDA was read using a plate reader with 488 nm excitation 525 nm emission (TECAN Infinite M1000 Pro, Männedorf, Switzerland). ROS concentration was estimated as the difference in fluorescence units per cell between the stained and unstained aliquots of each culture. This metric was also divided by ESD size to account for size effects.

### Taxonomic confirmation of strains

DNA from stock cultures (10 mL) was extracted using a DNeasy PowerSoil kit (QIAGEN Inc., CA, USA) and checked for quality with a NanopDrop™ 2000 (ThermoFIsher Scientific, MA, USA), before amplification and sequencing at the Australian Genome Research Facility (AGRF, Sydney, Australia). PCR conditions and primers used were those developed by Chappell et al. [52] for the ITS region: forward primer: 5ʹ-RCGAAYTGCAGAACCTCG-3ʹ, reverse primer: 5ʹ-TACTYAATCTGAGATYCA-3ʹ.

Bioinformatics processing was conducted using Geneious Prime (Version 2020.0.5; Biomatters Ltd.). Strain sequences were compared to GenBank using the BLAST function to confirm species identity. Nucleotide sequences were aligned using the MUSCLE alignment [53], followed by Bayesian inference analysis using MrBayes [54] to generate a phylogenetic tree. The out-group for the tree was a strain of *Chaetoceros atlanticus* isolate TPV2 1146 obtained from GenBank. Percentage similarity between strains according to the alignment was used as a metric of genetic relatedness.

### Statistical analysis

We assessed the multivariate phenotypes for the *Thalassiosira* strains using principal component analysis (PCA). The input variables were the 9 independent trait measurements made on each replicate culture (*n* = 36, 3 biological replicates per strain). Trait data was standardized (mean = 0, SD = 1) for each trait prior to PCA analysis to account for differences in the units and scale of measurements. The resulting PCA plot was defined as the ‘trait-scape’.

Hierarchical clustering analysis was performed on the 9-trait dataset used to assess similarity in multivariate phenotypes between each replicate for each strain (*n* = 3 per strain).

To compare genetic vs. phenotypic similarity, percentage similarity between strains was correlated against the distance between strain centroids (multivariate means) within the trait-scape. Distances between multivariate means (centroids) were calculated using the equation:

$${{{{{{{\mathrm{distance}}}}}}}} = \sqrt {\left( {{{{{{{{\mathrm{{\Delta}}}}}}}PC}}1.{{{{{{{\mathrm{a}}}}}}}}} \right)^2\,+\,\left( {{{{{{{{\mathrm{{\Delta}}}}}}}PC}}2.{{{{{{{\mathrm{b}}}}}}}}} \right)^2}$$

ΔPC1 is the difference in PC1 co-ordinates between the two strains, a is the % variance explained by PC1, ΔPC2 is the difference in PC2 co-ordinates between the two strains, b is the % variance explained by PC2.

To assess whether a trait-scape generated using fewer input traits (4 rather than 9) was representative of the full, 9-trait plot, we conducted PCA using 4 input traits, and then assessed whether the inter-strain distances (distances between centroids) within the plot were correlated using linear regression. This provided a quantitative assessment of whether the strains were in the same relative positions to each other within the trait-scape.

### Covariation of traits

To compare the pairwise relationships between traits across the strains, correlation matrices were made using data collected in the standard environment, and for the HT and LN environments.

### Phenotypic plasticity

The change in phenotypes in the new environments were assessed firstly by conducting PCA on the full dataset, including trait data from the 13 strains grown in the standard environment, plus the 5 strains grown in the two additional environments. This generated an “expanded trait-scape”. In addition, correlation matrices were generated for the new environments' trait dataset to assess differences in trait-trait relationships between the ‘standard’ and “expanded” datasets.

Relative changes in trait values for each trait in the new environments were calculated as follows:

$$	{{{{{{{\mathrm{Relative}}}}}}}}\,{{{{{{{\mathrm{change}}}}}}}} \\ 	= \frac{{{{{{{{{\mathrm{trait}}}}}}}}\,{{{{{{{\mathrm{value}}}}}}}}\,{{{{{{{\mathrm{new}}}}}}}}\,{{{{{{{\mathrm{environment}}}}}}}} - \overline {{{{{{{\mathrm{x}}}}}}}} \,\,{{{{{{{\mathrm{trait}}}}}}}}\,{{{{{{{\mathrm{value}}}}}}}}\,{{{{{{{\mathrm{standard}}}}}}}}\,{{{{{{{\mathrm{environment}}}}}}}}}}{{\overline {{{{{{{\mathrm{x}}}}}}}} \,\,{{{{{{{\mathrm{trait}}}}}}}}\,{{{{{{{\mathrm{value}}}}}}}}\,{{{{{{{\mathrm{standard}}}}}}}}\,{{{{{{{\mathrm{environment}}}}}}}}}}$$

We used PCA to assess whether the relative changes in trait values were consistent between strains in the two different environments. i.e., was the relative change in whole phenotype consistent. If the changes were consistent across strains, we expected to see clustering in the PCA based on treatment.

### Statistical software

Statistical analyses were performed in R [55], Matlab, and Microsoft Excel. Hierarchical clustering analysis with multiscale bootstrap resampling (1000 replicates) on trait values from biological replicates was done with the ‘pvclust’ package in R [56] using Euclidean distance and the average (UPGMA) method. Principal component analysis was used to generate the multivariate trait-scape was done using the “vegan package” in R [57]. The contributions of each trait to the PC axes (loadings) were extracted using the “factoextra” package in R [58]. Trait correlation matrices were generated using the “corrplot” package in R [59].

## Results

### Defining the Thalassiosira trait-scape

**Fig. 1:**
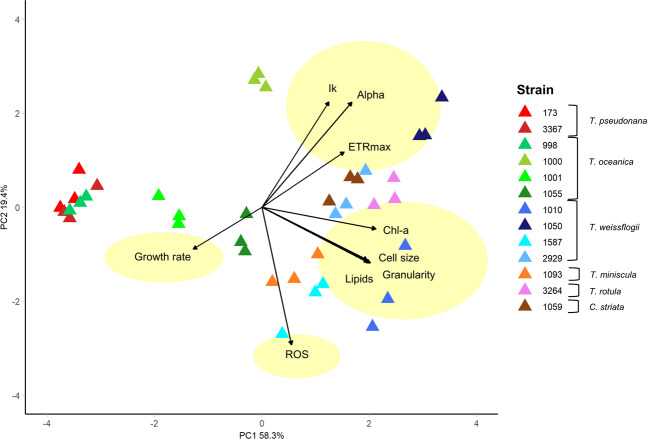
The multivariate phenotypic trait-scape of *Thalassiosira*. The trait-scape was determined using a principal component analysis generated using 9 input traits (growth rate, Ik, alpha, ETRmax, Chl-a, cell size, granularity, lipids, and ROS). Symbol colours represent different strains grown in triplicate. Yellow ovals represent the four groups of input traits as determined through trait correlations and contributions to the principal component axes.

To define the trait-scape for *Thalassiosira*, we measured nine traits in 13 taxa (Supplementary Table 1) in a standard nutrient rich environment. The principal component analysis of these 9 traits showed that two orthogonal axes (the first two principal components) captured a total of 77.7% of the variation between *Thalassiosira* strains (Fig. 1; Table 1). If trait values were completely randomly distributed in the trait-scape, only 25% (=2/8) of trait variation would be expected to be captured on two axes. This suggests that there are multi-dimensional relationships between the measured traits and that reduced dimensionality trait-axes can be used to understand *Thalassiosira* phenotypes. Below we identify the observed trait relationships, demonstrate the range of phenotypes present amongst *Thalassiosira* strains, and highlight the difference between inter- and intraspecific variations in trait values.

**Table 1:** Summary of the principal component analyses for the *Thalassiosira* trait-scape.

	Original trait-scape			Expanded trait-scape		
	PC1	PC2	PC3	PC1	PC2	PC3
Standard deviation	2.29	1.32	0.86	1.97	1.46	1.28
Proportion of variance	0.58	0.19	0.08	0.43	0.23	0.18
Cumulative proportion	0.58	0.78	0.86	0.43	0.67	0.85
Trait contributions						
Growth rate	6.54	3.18	70.28	5.35	2.02	29.09
Alpha	9.41	5.53	4.49	3.28	10.65	28.3
ETRmax	11.22	20	1.28	3.87	38.91	0.04
Ik	6.24	20.13	11.54	1.1	25.09	15.93
ROS	1.23	34.25	1.89	3.86	5.13	23.72
Cell size	15.41	5	6.75	23.21	0.29	2.11
Chlorophyll a	18.03	0.85	0.85	21.02	3.38	0.68
Lipids	15.56	5.47	0.6	15.29	11.59	0.08
Granularity	16.34	5.6	2.31	23.03	2.93	0.03

Relative contributions to the first three axes of the principal component analyses of 9 phenotypic traits for 13 strains of *Thalassiosira* grown in the standard environment (original trait-scape), then including the high temperature/light and low nutrient conditions (expanded trait-scape).

### Co-variance of traits across Thalassiosira strains

**Fig. 2:**
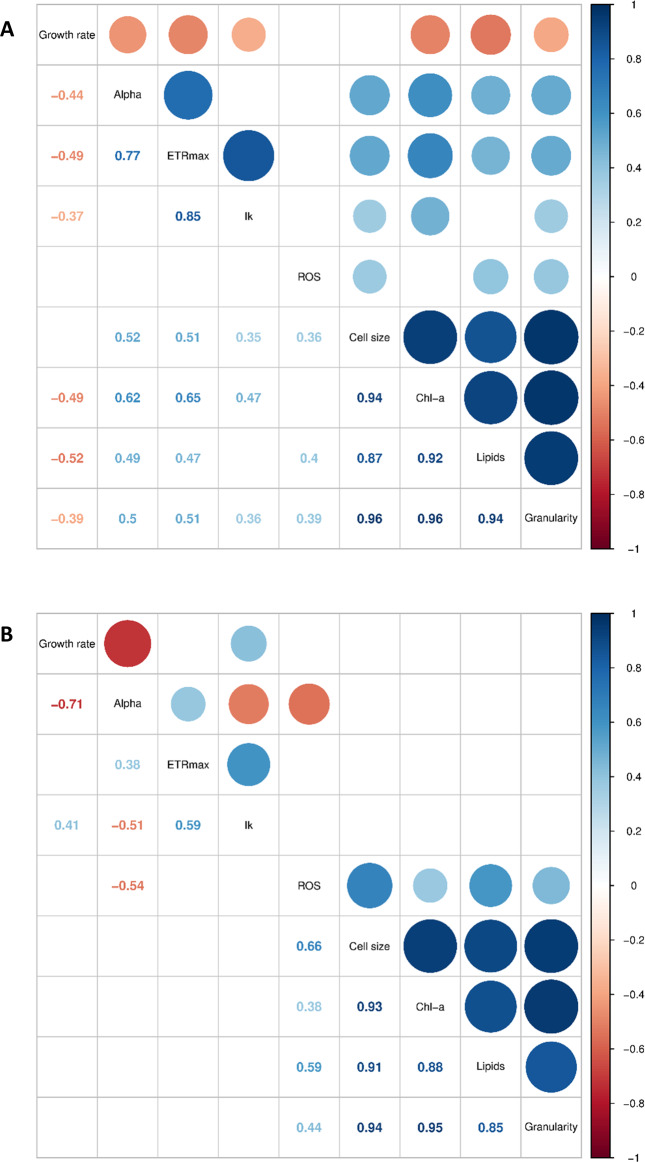
Correlation matrices of trait data for *Thalassiosira* strains grown under standard vs. stressful growth conditions. **A** 13 strains under standard growth conditions (f/2 media, 20 °C, 60 µmol photons m^−2^s^−1^ light) and (**B**) 5 strains grown under high temperature/light (f/2 media, 30 °C, 200 µmol photons m^−2^s^−1^ light) and low nutrient (f/400 media with adjusted N:P ratio of 10:1, 20 °C, 60 µmol photons m^−2^s^−1^ light) treatments. Displayed values are Pearson’s linear correlations between trait combinations with significance of *P* < 0.05.

Strong, consistent relationships were observed between certain traits across all 13 strains. Specifically, cell size, granularity, Chl-a, and lipid content were all positively correlated with one another and were all highly correlated with PC1, which accounted for 58.3% of the total variation (Fig. 2A, Pearson’s *R*^2^ > 0.87, *P* < 0.05; Table 1). This indicates that these traits are the key explanatory variables of differences between *Thalassiosira* strains investigated in this study. Cell size in the standard environment ranged from 4.2 to 16.1 µm, with the smallest celled strains (<5 µm: 3367, 178, and 998) showing higher growth rates and similar overall phenotypes (Fig. 1 and Supplementary Figs. 1 and 2). However, across all taxa, there was no significant correlation between growth rate and size. In addition, growth rate did not contribute strongly to either of the first two principal component (PC) axes. This demonstrates that there are multiple combinations of the traits across the 13 strains that produce similar growth rates (Supplementary Fig. 2). As cell size increased (towards the right side of the trait-scape), other aspects of the phenotypes showed more variability between strains, indicated by increased dispersion of strains on the PC2 axis (Fig. 1; PC2 coordinates ranged from −2.70 to 2.55 when PC1 > 0, compared to −0.93 to 2.84 when PC1 < 0). PC2 primarily represents ROS production, ETRmax, and Ik (Table 1). This indicates that, as size increases, other traits tend to become more variable (less predictable).

### Strains’ distribution within the trait-scape

**Fig. 3:**
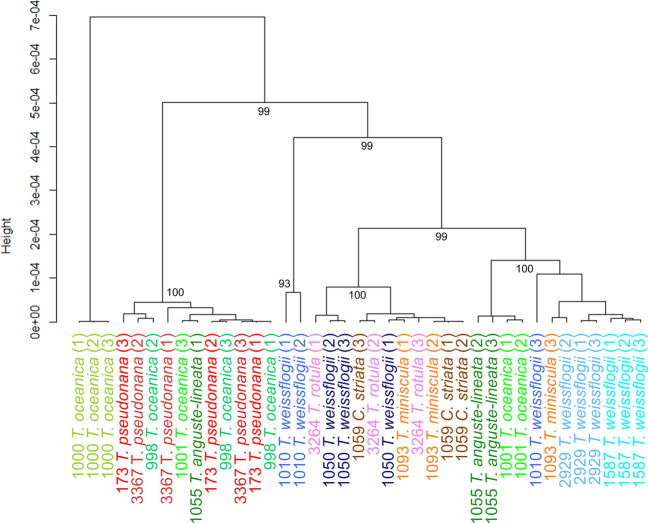
Hierarchical clustering of multivariate phenotypes for 13 *Thalassiosira* strains. Hierarchical clustering analysis of multivariate phenotypes for each 13 *Thalassiosira* strains grown in the standard environment (*n* = 3). Labels are strain code followed by species and replicate number. Black values indicate the approximately unbiased (AU) *p* value for non-selective inference from multiscale bootstrap resampling (*n* = 10,000). Strain 1000 comprises its own cluster, with the other strains split into two other main clusters: one comprising small cell strains (173, 3367, 998, and one replicate of 1001) and the other comprising all other strains. Smaller subgroups were also found within the three larger groups, AU *p* values >95 indicate that these smaller clusters are strongly supported by the data.

The trait-scape defined by the two PC axes was not uniformly occupied, with strains showing evidence of clustering (Figs. 1 and 3). Strain 1000 was distinct from all other strains, having differing photophysiology (higher ETRmax, alpha and Ik values), higher ROS accumulation, and slower growth than other similar-sized strains. This may indicate that this strain was experiencing more stress than other strains under these conditions. Within the rest of the trait-scape, there was one distinctive broader phenotypic cluster that encompassed multiple species: the “small-celled” strains (Figs. 1 and 3; strains 998, 3367 and 173; mean cell sizes 4.1–4.7 µm).

### Genetics vs. phenotypic similarity between strains

Comparison of the ITS2 gene region showed that strains of the same species shared the highest sequence similarity, as would be expected (Supplementary Fig. 3). However, there was no relationship between genetic relatedness and similarity in phenotype, with the percentage ITS2 sequence similarity being unrelated to proximity of strains in the trait-scape (*R*^2^ = 0.05, *P* > 0.05 Supplementary fig. 4). Hierarchical clustering also showed that phenotypes were not discretely grouped by genotype (Fig. 3).

### Recovery of the trait-scape with a reduced number of traits

**Fig. 4:**
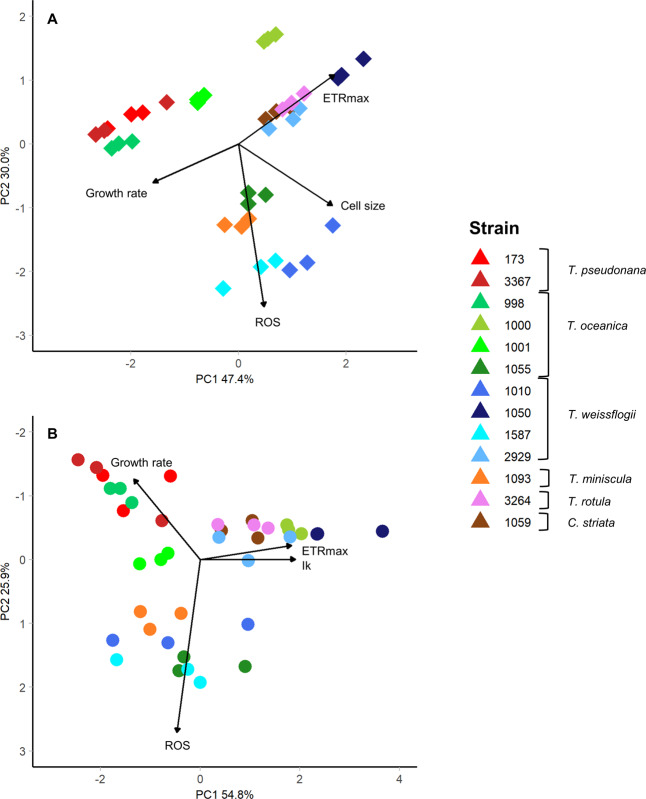
The multivariate phenotypic trait-scape of *Thalassiosira* generated with a reduced numbers of traits. The original trait-scape (from 9 input traits) was re-created using principal component analysis of (**A**) 4 input traits representing the four trait groups (growth rate, cell size, ROS, ETRmax). **B** 4 input traits representing three out of the four trait groups, excluding cell size-related traits (growth rate, ROS, ETRmax, Ik).

A key aim of this study was to assess how complex multi-trait phenotypes can be understood using reduced dimensionality axes. Here we assess whether a similar phenotypic trait-scape can be resolved using fewer traits. The PCA identified four orthogonal trait ‘groups’ within the trait-scape (Fig. 1 yellow circles). The two main groups were the cell size-related traits and photosynthesis-related traits which weighted strongly on PC1 and PC2, respectively (Fig. 1; Table 1). ROS and growth rate were included as orthogonal, independent traits due to their lack of correlation with the two main trait groups (Fig. 2A). We were able to capture the same distribution of phenotypes (trait-scape) using a reduced number of traits as the full set of traits, (Fig. 4A), with the first two PC axes capturing 70.4% of the variance between strains (Table 1). This demonstrates that a reduced-input analysis for these traits can adequately represent the similarity between strains observed in the higher resolution trait-scape (Adjusted *R*^2^ = 0.81, *P* < 0.05; Supplementary Fig. 5), without losing the ability to distinguish different taxa.

To test the robustness of this results, we repeated this analysis with four input traits representing 3 of the 4 trait groups (i.e. no cell size-related traits). This resulted in a trait-scape that less accurately resembled the original trait-scape (Fig. 4B), with inter-strain distances that were only weakly correlated with the distances in the original trait-scape (Adjusted *R*^2^ = 0.30, *P* < 0.05; Supplementary fig. 5). Although growth rate differences were not important in distinguishing between strains, we kept growth rate in our analysis because it determines population size and is often used as a fitness proxy. We tested the removal of growth rate from the trait-scape and found that three remaining trait groups captured 61.9% and 21.4% of the variance on 2 axes (83.3% total). It is important to note, that this analysis does not suggest that cell size and photosynthetic traits are necessarily universal ‘master’ traits but rather that, for *Thalassiosira*, these are critical trait groups necessary for understanding phenotypic diversity. Moreover, robust reduced dimensionality phenotypic axes (trait-scape) can be created with a curated set of traits thus facilitating experimental and modelling studies.

### Multivariate-trait plasticity

Interspecific relationships are often used to predict phenotypic changes under different environmental conditions. Here we test the robustness of this assumption. A subset of five strains encompassing diverse phenotypes were selected and grown in two new environments (high temperature and light; low nutrients; Supplementary fig. 6). These environments were selected as they are known to induce plastic responses (short-term change in at least one trait value). The full suite of 9 traits were then quantified in the new environment and the shift in phenotypes was assessed.

**Fig. 5:**
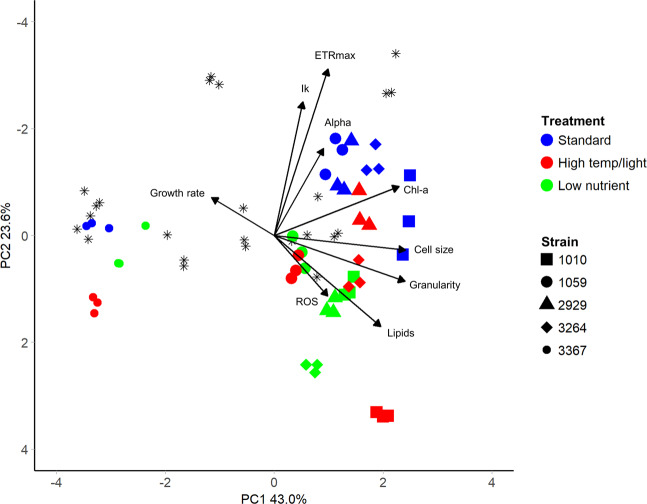
The expanded multivariate phenotypic trait-scape of *Thalassiosira*. The principal component analysis was done using trait data from the standard environment (13 strains) and the strain subset grown in two additional environments (5 strains). The 9 input traits were growth rate, cell size, Chl-a, granularity, lipids, ROS, Ik, alpha, ETRmax. Black stars indicate phenotypes from the strains grown only in the standard environment (n = 8), from the original trait-scape. Coloured points are the subset of 5 strains (shapes) grown in the standard environment (blue), high temperature/light environment (red), and low nutrient environment (green).

Both new environments resulted in trait combinations not observed under standard replete conditions and increased the range of measured trait values (Fig. 5). In the expanded *Thalassiosira* trait-scape, the first two principal components captured 66.6% of the phenotypic variation (Fig. 5). The expanded trait-scape contained the same two primary trait groups and similar weightings of each trait on the PC axes as the original trait-scape, with cell size-related traits correlating the most to PC1, and the photophysiological traits correlating to PC2 (Table 1). With the addition of more stressful environments, ROS switched from contributing significantly to PC2 (34.25%) to explaining minimal variance on both PC1 (3.9%) and PC2 (5.1%) in the expanded trait-scape (Table 1). The inclusion of data from the new environments did not significantly alter the observed trait correlations (Fig. 2B), which is consistent with the idea that trait correlations are relatively constrained for a given set of genotypes over many environments, and therefore limit the number of possible expressed phenotypes.

We utilized the trait-scape to assess the phenotypic movement of each strain (phenotypic plasticity). For all strains, the phenotypes resulting from plastic responses to the new environments were more separated from the ‘standard’ environment along PC2 (Fig. 5). Differences along PC1 were less pronounced and not consistent in direction across strains or environments (Fig. 5). This illustrates greater phenotypic plasticity in the photophysiological traits (highly correlated with PC2), rather than cell size-based traits amongst the taxa. In some instances, the intra-species variability in the expanded trait-scape exceeded the inter-species variability (distances between strains) from the standard environment (Fig. 5). In other words, the range of observed trait combinations in the new environments exceeded the observed interspecific variability, even though the relationships between the traits remained relatively stable.

Cell size and related traits in the new environments were all positively correlated, as in the standard environment (all *R*^2^ ≥ 0.85, *P* < 0.05; Fig. 2B). ROS was significantly correlated with cell size traits but the relationships were not all strong (*R*^2^ ≤ 0.66, *P* < 0.05; Fig. 2B). Growth rate was not strongly correlated with any other trait, as in the standard environment, with the exception of a negative relationship with alpha (Fig. 2B), however neither growth rate nor alpha contributed significantly to the two main PC axes (Table 1). Within the photophysiological traits, the strong correlations between ETRmax and alpha, and ETRmax and Ik observed in the original trait-scape were weakened in the new environments (Fig. 2A and B).

**Fig. 6:**
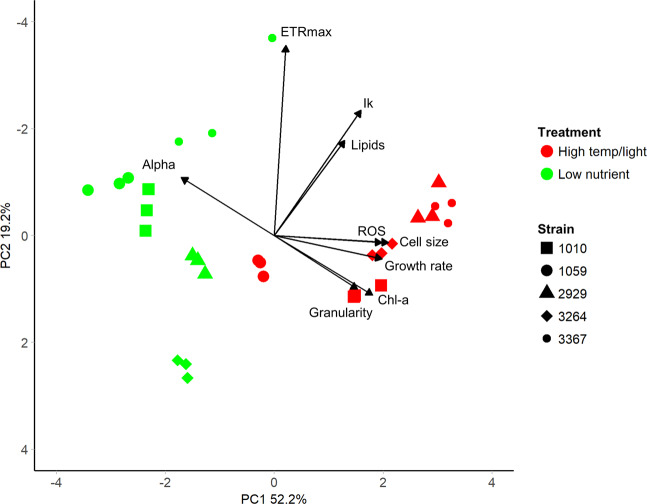
Phenotype shifts of *Thalassiosira* strains grown in new environments. Principal component analysis of the relative trait changes between the standard environment and the high temperature/light (red) and low nutrient (green) environments for the subset of five strains (shapes). Black stars are the remaining strains grown only the standard environment (*n* = 8).

The relative changes in trait values for each strain in the new environments were analysed together using PCA (Fig. 6). Within the PCA plot, strains are grouped according to environment, indicating that the overall change in phenotype in response to the new environments (i.e., the combination of changes in all of the different traits) was conserved across all strains.

These two groups of responses could be classified as “strategies”, changes in the multivariate phenotype which encompass n-directional trade-offs and correlations between individual traits. These observations show us that regardless of the standing diversity in the standard conditions (Fig. 1), these strains utilise the same strategies in response to environmental change (Fig. 6), and that trait correlations constrain which phenotypes are accessible as plastic responses. This analysis shows that the relative changes in traits were grouped according to the novel environment.

## Discussion

In this study, we used multivariate methods to define a trait-scape for the marine diatom genus *Thalassiosira*. By reducing the dimensionality of a 9 trait dataset to two orthogonal axes, we demonstrate how this statistical framework can be used to examine multi-trait phenotypes and changes to those phenotypes in new environments. Our study shows that underlying variation between phytoplankton phenotypes can be explained by a reduced number of principal component axes, and that those axes can be reproduced using a reduced number of traits. Such reduced dimensionality within species has been described in invertebrates [19], and other microalgae [35], and suggests that, due to trade-offs, there are not infinite numbers of possible trait combinations that organisms can manifest. The result of this is that diatom trait-scapes are not uniformly filled and certain areas represent physiologically impossible trait combinations.

The implication of constraints in how trait-scapes are filled is significant: constrained variation among phenotypes potentially makes representing responses in biogeochemical and ecosystem models more tractable. In principle, there are an infinite number of trait combinations and trait-scapes that could be measured on a large number of diverse phytoplankton taxa [10]. However, studies of phytoplankton are generally interested in a fairly well constrained list of traits that affect their roles in food webs, nutrient cycles, and biogeochemistry. Our results indicate that numerous strains are necessary to quantify underlying phenotypic diversity, but that relatively few strains of *Thalassiosia* may be used to identify divergent strategies in different environments. Furthermore, our results suggest that among phytoplankton traits commonly used in ecosystem models, a reduced number of key traits and their relationships can explain overarching phenotypic responses to environmental change, potentially allowing more informative experiments to be designed for understanding such biological responses. Designing such experiments requires initial consideration of which traits to measure (based on orthogonality within the trait-scape), as well as choosing a sufficient, but not exhaustive, number of genotypes. By reducing the number of traits and genotypes, experimental resources can be focussed on testing multi-trait responses to different environments, which is essential for understanding the degree of plasticity within and between species.

### Phenotype/Genotype relationships

For *Thalassiosira*, phenotypic diversity could not be predicted from genotypic diversity or taxonomic classification, and plasticity created divergent phenotypes that did not cluster according to genetic similarity. This suggests that estimates of trait diversity based on sequence variation are unlikely to be accurate for closely related organisms (i.e., within species), as evidenced in freshwater chlorophytes [60]. Trait-based ecology typically assumes intraspecific variation to be lower than interspecific variation [1], but our study adds to the growing body of evidence that inter and intraspecific diversity plus diversity resulting from plasticity can be substantial in phytoplankton [13, 35, 61–63]. Notably, phenotypic plasticity is generally unaccounted for in species distribution and biogeochemical models, even though it is recognised that plasticity can significantly affect model estimates [64].

Although the uncoupling of genotype and phenotype indicates a cautionary tale for making assumptions about any strain or species’ phenotype based on genotype, the constraints demonstrated in the trait-scape show that there are underlying rules of phenotypic expression and plasticity that hold across genotypes. This has the potential to simplify ecological modelling by constraining the trait value combinations available within species or broadly similar taxa, and can inform models of longer-term (evolutionary) responses [65].

### Multivariate trait trade-offs are more complex than 2 or 3 traits

To understand and model phytoplankton responses to environmental change, we need to consider how complex phenotypes vary in response to environmental perturbations. This requires a multi-dimensional representation of traits, incorporating not only pairwise, but multi-dimensional trade-offs within and between species. However, there is a paucity of evidence of multi-directional trade-offs in the phytoplankton, and an even greater lack of empirical data, partially due to the daunting logistics of carrying out the necessary experiments, as well as the challenges of representing the results in a meaningful way.

Here we demonstrate that the constraints on the phenotype in response to environmental change can be described in terms of multivariate trade-offs (Fig. 5), that may not have been apparent by looking at only two traits at a time. By using a “trait-scape” approach we can assess multi-trait phenotypes simultaneously rather than presenting multiple two-way correlations which could miss critical underlying multi-trait relationships. Movement within the trait-scape incorporates the n-dimensional intra-specific trade-offs occurring between our 9 traits and represents holistic “strategies” (Fig. 6) that may be conserved across functional groups. Constraints on phenotypic shifts that can be expressed in response to environmental change limit the number of overall strategies available, even with different genetic starting points [66, 67]. Our observations also suggest that trait-scapes defined using ecologically important traits are not uniformly occupied and that multi-directional trade-offs can be used to define the bounds of accessible phenotypes for *Thalassiosira*. One direction for future work would be to determine the limits of genetic distance over which these apparent constraints hold.

### Across-species vs. short-term physiological trade-offs are different

**Fig. 7:**
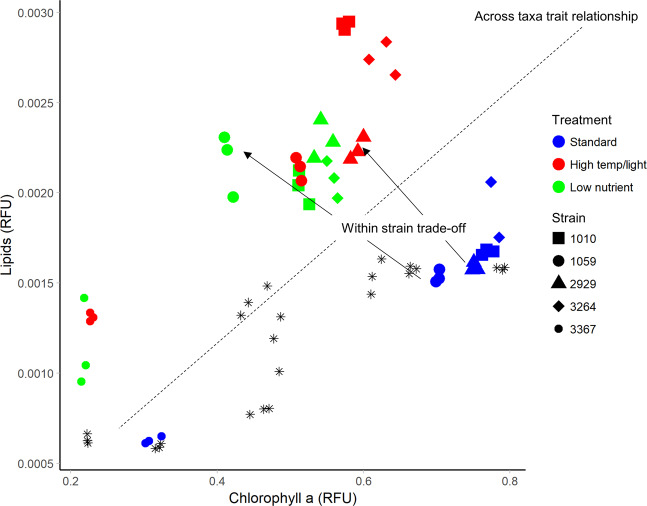
Trait-trait relationship between chlorophyll a and lipid content between and within *Thalassiosira* strains. Blue, green, and red symbols show the subset of strains (*n* = 5) grown in the three different environments: standard, high temperature/light, and low nutrients. Black stars are the remaining strains grown only the standard environment (*n* = 8). The overall correlation between these two traits across taxa was positive, however in the new environments, a negative trade-off occurs as lipid content increases with a decrease in chlorophyll a. This occurred in all five strains.

Trait correlations observed in multiple strains or species, such as cell size and growth rate when considered across diatoms, dinoflagellates, cyanobacteria etc. [68], are generally interpreted as fundamental trait trade-offs that are unlikely to evolve. However, there is an important distinction to be made between trade-offs seen across species/lineages, and constrained plastic responses within a lineage but across environments [21]. Within a population, these relationships may not follow wider trade-off patterns, and instead represent a short-term physiological trade-off. For example, in our study, Chl-a and lipid content were positively correlated when viewed across all taxa (Fig. 7). In contrast, within any one strain grown under low nutrients, cells reduced their Chl-a content but increased lipid content, implying a possible short-term trade-off between lipid and Chl-a synthesis (Fig. 7). Under nutrient limitation or other stress, many microalgae upregulate neutral lipid production and storage as a means to store energy [69]; indeed *Thalassiosira pseudonana* cells can modify their lipid composition and increase lipid stores within as little as 24 h after a reduction in nitrogen availability [70]. Conversely, nutrient limitation can decrease chlorophyll content of *Thalassiosira* in the presence of high CO_2_ [71].

The distinction between across-species and short-term physiological trade-offs is a challenge for modelling applications as the “rules” for how phytoplankton respond to short term changes may not be reflected by trait correlations resulting from longer term evolution. Our results suggest that further studies to explore short-term physiological trade-offs through experimentation are required to determine the limits and patterns of phenotypic plasticity, which can influence both the speed and direction of evolutionary processes [72].

While we kept our culturing processes consistent across all strains to minimise any phenotypic variance due to cultivation factors, assessing the contribution of evolutionary or culturing history to phenotypic responses was beyond the scope of this study. Our observation of *Thalassiosira* taxa in extended culture show that vessel type and time in cultivation can affect trait values [42] but confirm that the phenotypic plasticity as quantified in this study was largely due to the environmental conditions imposed on strains/species in our experiments.

### How will diatoms fare in the future ocean?

Plastic and adaptive responses will play a key role in how diatoms fare in response to climate change, as the frequency of extreme events and overall variability in the oceanic environment increases [73]. Diatoms are one of the most diverse groups of organisms in the ocean with a large number of taxa [74], and high genetic diversity within populations [75–77]. This diversity creates a challenge for making predictions about how diatoms will respond to a changing ocean. The trait-scapes and trait relationships presented in this study provide a framework for generating and testing hypotheses about constraints on the number of possible phenotypes at the genus, family and potentially phytoplankton functional group level. The genetic distance over which these constraints hold remains to be tested, but we suggest that collapsing multiple traits onto a reduced set of orthogonal axes could assist with predictions of future phytoplankton communities either by direct inclusion of traits and their correlations into models, or through consideration of plastic or evolutionary trajectories within the trait-scape [34].

## Supplementary information

Supplementary Information
