# Supplementary material for: Multivariate trait analysis reveals diatom plasticity constrained to a reduced set of biological axes
Source: ISME Commun. 2021 Oct 25;1:59. doi: 10.1038/s43705-021-00062-8 (PMC9723791; doi:10.1038/s43705-021-00062-8)
Supplement: Supplementary file 1 — Supplementary Information [file 43705_2021_62_MOESM1_ESM.pdf]

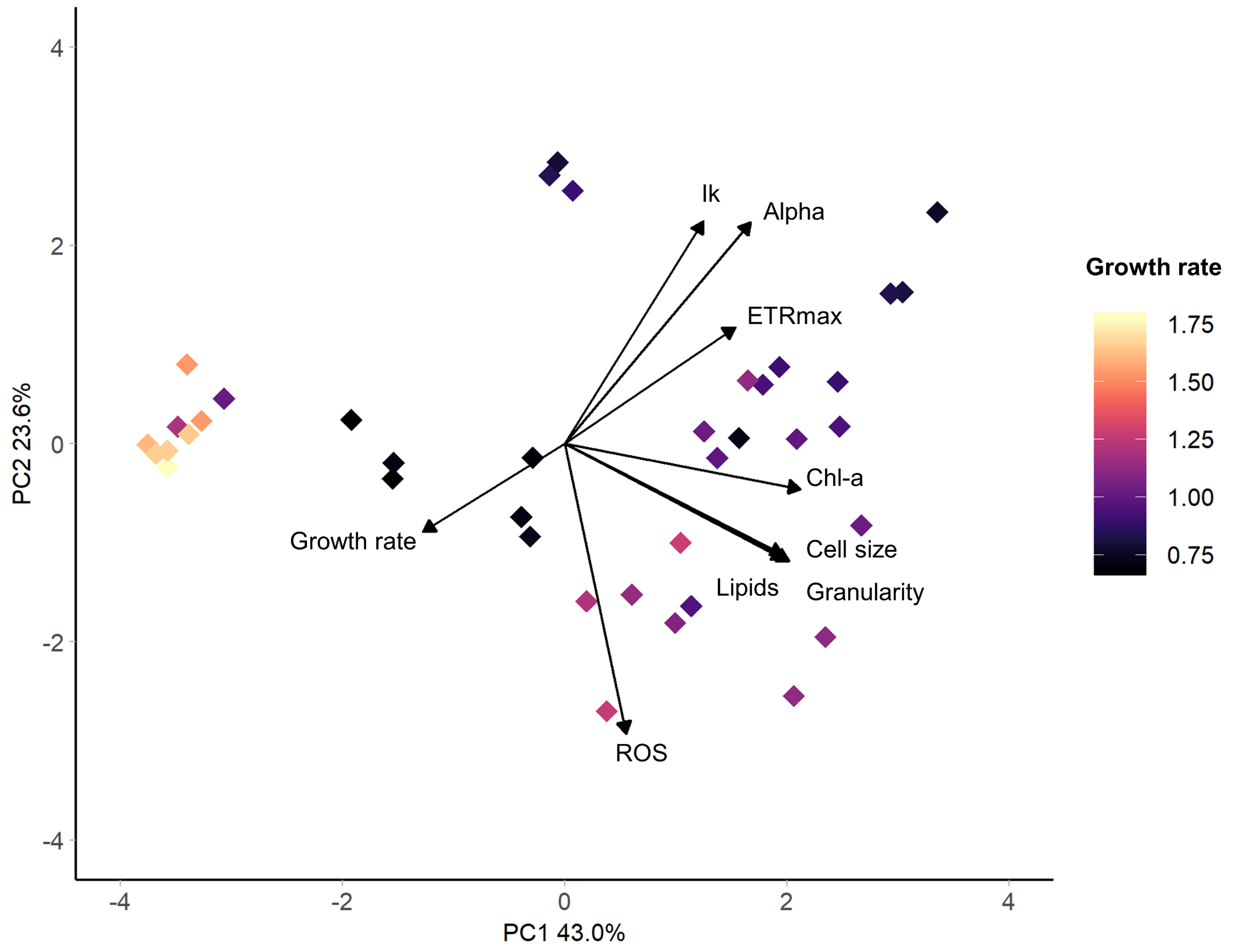

Supplementary Figure 1. The *Thalassiosira* trait-scape from the standard environment visualised by growth rate. This is the same figure as Fig 1, however each point is coloured according to growth rate. Faster growing cells to the left hand side represent the smaller strains, as cell size is positively correlated with PC1.

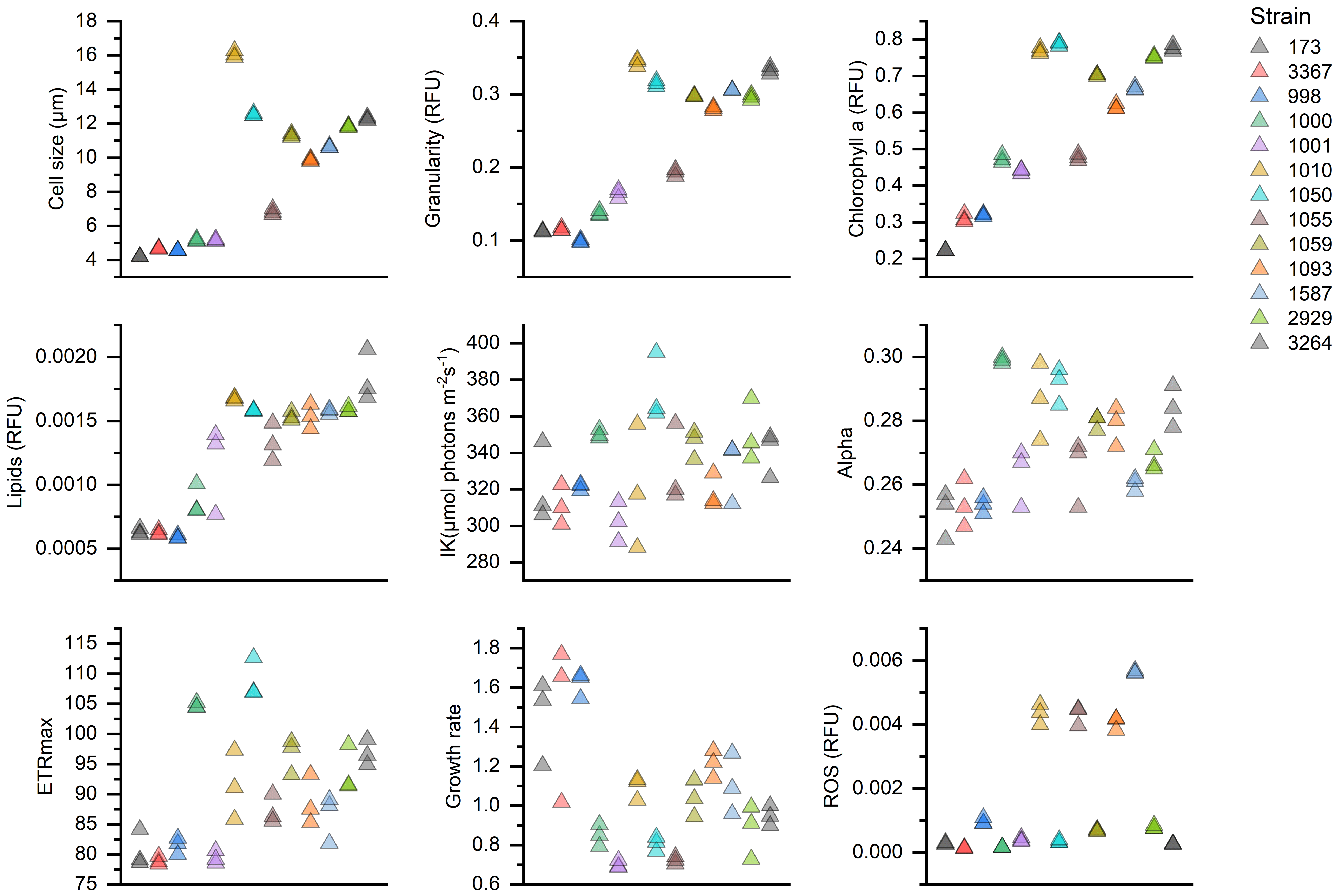

Supplementary Figure 2. Raw trait values measured on *Thalassiosira* strains. Raw trait values of 9 phenotypic traits measured in this study on 13 strains of *Thalassiosira* (n=3). Strains are shown in different colours. Values shown for cell size-related traits granularity, chlorophyll a, and lipids have all been corrected for size effects by dividing the raw output value by the cell size value (representing equivalent spherical diameter).

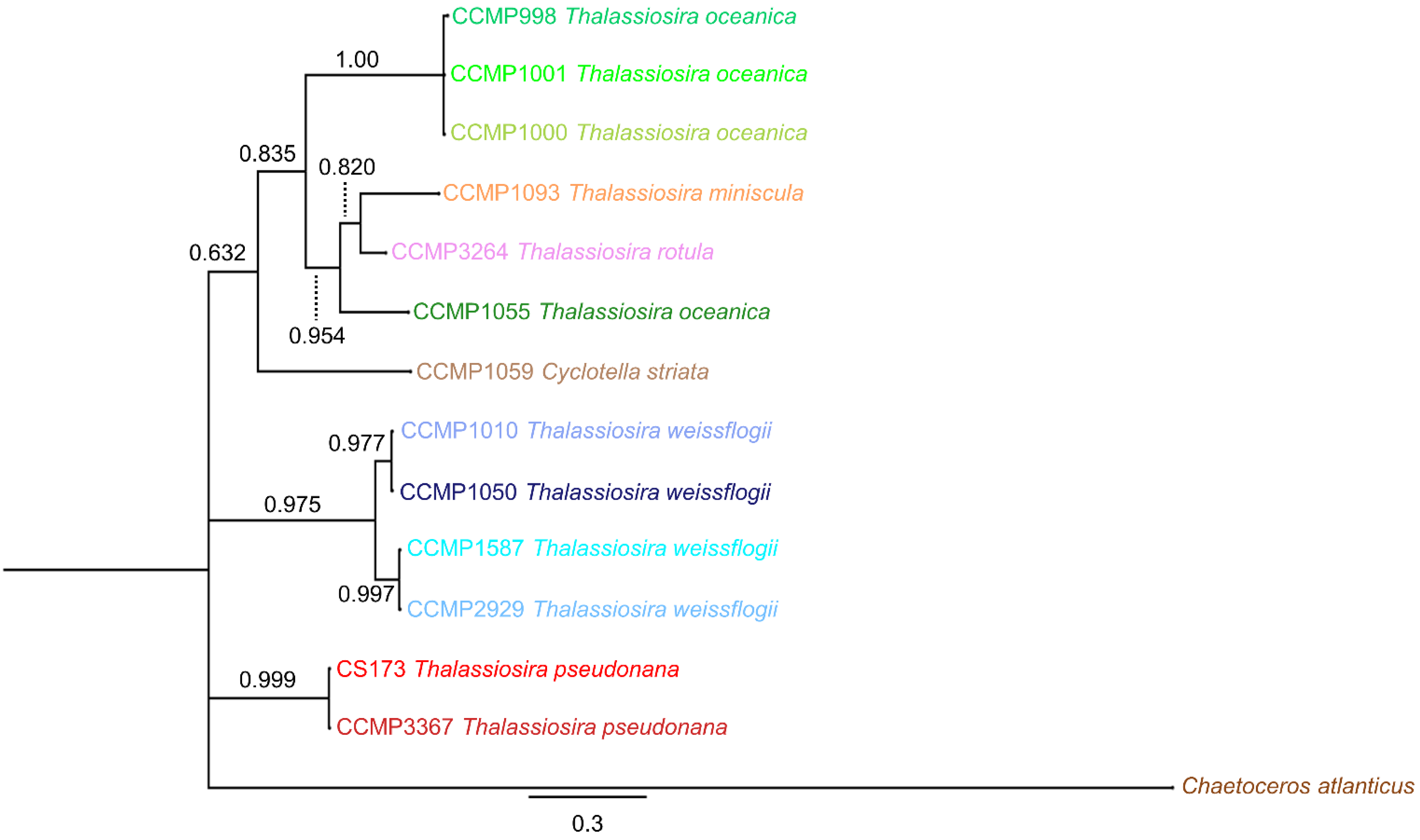

Supplementary Figure 3. Genetic relatedness of the 13 *Thalassiosira* strains based on ITS2 sequencing. Phylogenetic relationships of the *Thalassiosira* strains (Bacillariophyceae) used in this study, based on Bayesian analysis of the ITS2 gene region. Node values indicate the Bayesian posterior probability support. The diatom *Chaetoceros atlanticus* was used as the root of the tree. Branch length indicates expected substitutions per site, i.e. the longer the branch, the greater the number of nucleotide substitutions.

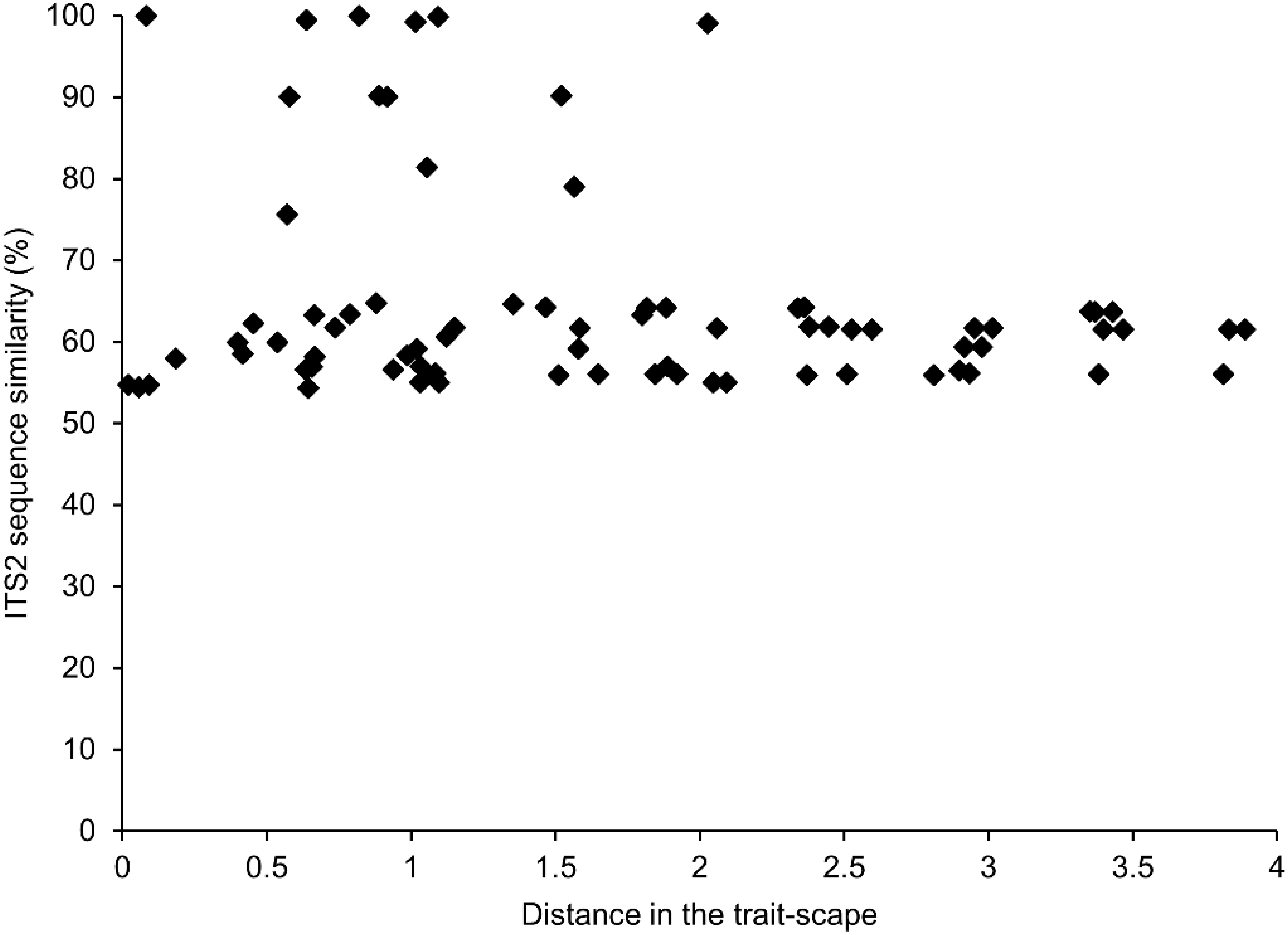

Supplementary Figure 4. Genotype – phenotype similarity between the 13 *Thalassiosira* strains. This plot shows the relationship between phenotypic similarity (distance between multivariate centroids in the trait-scape) and genetic similarity (percentage similarity of the ITS2 gene region sequences) between *Thalassiosira* grown in the standard environment. A positive linear correlation would be expected if phenotypic and genotypic differences between the strains were similar, but this was not the case for these data.

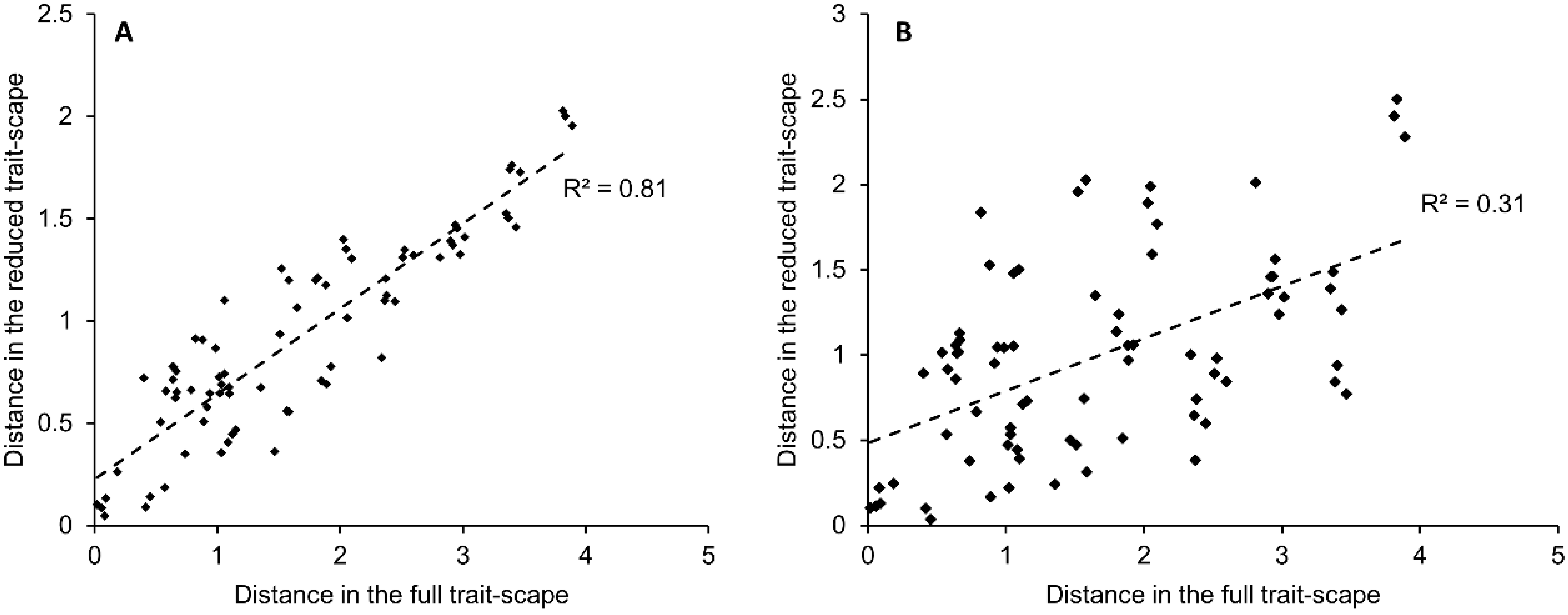

Supplementary Figure 5. Robustness of reduced-trait trait-scapes for *Thalassiosira*. The relationship between inter-strain distances in 'full' trait-scape generated with 9 input traits and **A** the reduced 4-trait including representative traits from all trait groups (growth rate, cell size, ROS, ETRmax) and **B** the reduced 4-trait-scape not including all trait groups (growth rate, ETRmax, Ik, ROS). In both plots Pearson's R^2 is shown for the linear correlation, with both being significant $P < 0.05$.

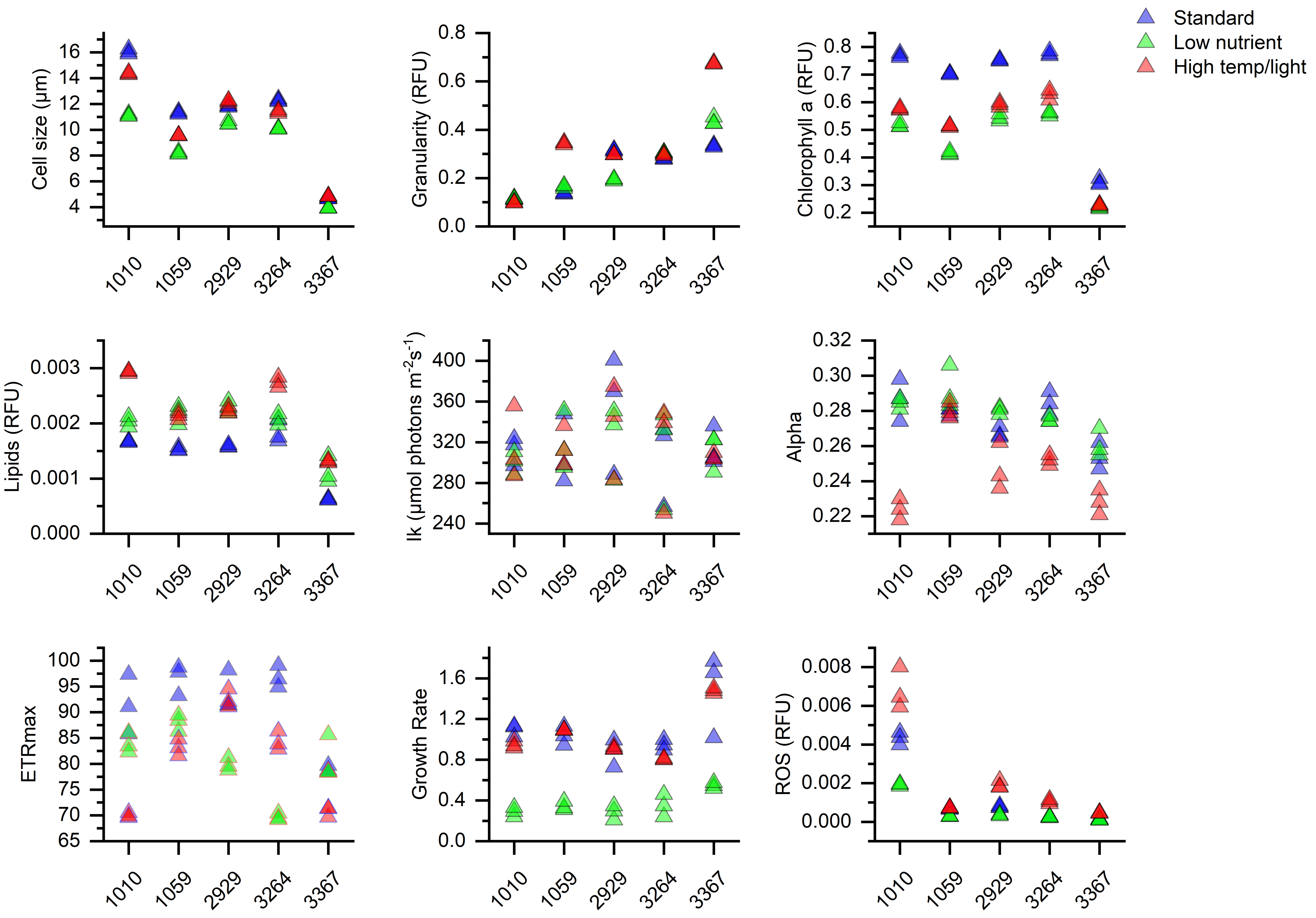

Supplementary Figure 6. Raw trait values of *Thalassiosira* strains grown in three environments. Raw trait values of 9 phenotypic traits measured in this study on 5 strains of *Thalassiosira* (n=3) grown in a standard(blue: f/2 media in artificial seawater with 20 °C 60 µmol photons $m^{-2}s^{-1}$ of light 12:12 light:dark), low nutrient (green: f/400 media with an adjusted N:P ratio of 10:1 achieved by reducing the nitrate concentration from 4.4 to 1.8 µM, 60 µmol photons $m^{-2}s^{-1}$ of light 12:12 light:dark), and high temperature/light (red: 30 °C with 200 µmol photons $m^{-2}s^{-1}$ of light 12:12 light:dark) environments. Strain codes are displayed along the x axis. Trait values for cell size-related traits granularity, chlorophyll a, and lipids have all been corrected for size effects by dividing the raw output value by the cell size value (representing equivalent spherical diameter).

Supplementary Table 1. Details of the 13 *Thalassiosira* strains used in this study. Species ID from culture collection, strain code, collection location, and identification based on a BLAST search of the ITS2 gene region sequences.

Species	Strain code	Collection location	Identification based on ITS-2 region
T. pseudonana	CS173	40.756° N 72.82° W Moriches Bay of the Forge River (Long Island, New York, USA)	*T. pseudonana*
T. pseudonana	CCMP3367	44.9335° N 12.7005° E North Adriatic Sea	*T. pseudonana*
T. oceanica	CCMP998	39.0833° N 71.9333° W North Atlantic	*T. oceanica*
T. oceanica	CCMP1000	39.0833° N 71.9333° W North Atlantic	*T. oceanica*
T. oceanica	CCMP1001	39.0833° N 71.9333° W North Atlantic	*T. oceanica*
T. oceanica	CCMP1055	33.1833° N 65.25° W North Atlantic	*T. oceanica*
T. weissflogii	CCMP1010	37° N 65° W, Gulf Stream, between Bermuda and New York (very approx)	*T. weissflogii*
T. weissflogii	CCMP1050	32.966° N 117.251° W, Del Mar Slough, California USA	*T. weissflogii*
T. weissflogii	CCMP1587	6.08° S 106.79° E , Jakarta Harbor, Indonesia (approx.)	*T. weissflogii*
T. minuscula	CCMP1093	32.9° N 117.255° W Scripps Institute of Oceanography pier, La Jolla CA	*T. miniscula*
T. rotula	CCMP3264	40.49° N 14.15° E, Marechiara, SZN long term sampling station, Gulf of Naples, Italy	*T. rotula*
Thalassiosira sp.	CCMP1059	19.665° N 156.034° W Aquaculture Pond, Oahu, Hawaii USA (approx.)	*Cyclotella striata*
Thalassiosira sp.	CCMP2929	Unknown	*T. weissflogii*

Supplementary Table 2. Duration of growth during the experiment for each *Thalassiosira* strain in the three different environments.

Environment	Species	Strain	Duration of active growth prior to trait measurements (days)
**Standard**	*T. pseudonana*	CS-173	9
	T. pseudonana	CCMP3367	8
	T. oceanica	CCMP998	8
	T. oceanica	CCMP1000	10
	T. oceanica	CCMP1001	10
	T. oceanica	CCMP1055	7
	T. weissflogii	CCMP1010	8
	T. weissflogii	CCMP1050	7
	T. weissflogii	CCMP1587	9
	T. minuscula	CCMP1093	8
	T. rotula	CCMP3264	8
	Thalassiosira sp.	CCMP1059	8
	Thalassiosira sp.	CCMP2929	9
**High temperature/light**			
	T. weissflogii	CCMP1010	3
	Thalassiosira sp.	CCMP1059	3
	Thalassiosira sp.	CCMP2929	3
	T. rotula	CCMP3264	4
	T. pseudonana	CCMP3367	5
**Low nutrient**			
	T. weissflogii	CCMP1010	5
	Thalassiosira sp.	CCMP1059	6
	Thalassiosira sp.	CCMP2929	6
	T. rotula	CCMP3264	5
	T. pseudonana	CCMP3367	6
